# Supplementary figures and images for: Exploring the Venom Gland Transcriptome of Bothrops asper and Bothrops jararaca: De Novo Assembly and Analysis of Novel Toxic Proteins
Source: Toxins (Basel). 2024 Nov 27;16(12):511. doi: 10.3390/toxins16120511 (PMC11728684; doi:10.3390/toxins16120511)

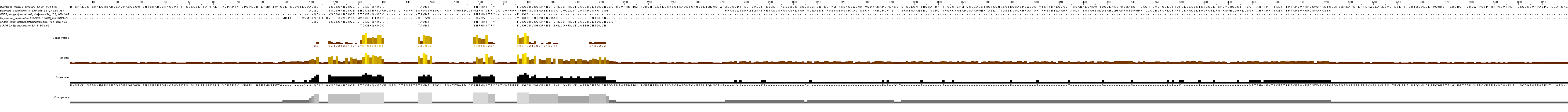

Supplement: Supplementary file 1 [file toxins-16-00511-s001.zip › toxins-3245296-supplementary/toxins-3245296-supplementary figures/Images/S10_alignment_Sntx3.png]

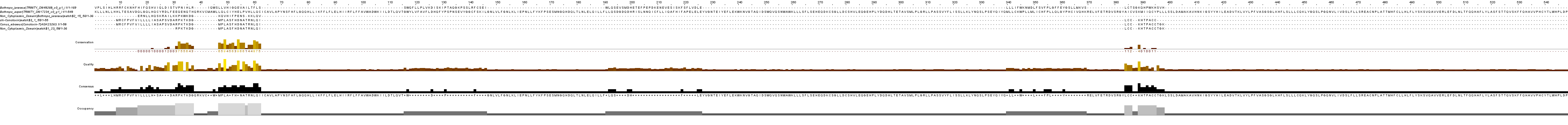

Supplement: Supplementary file 1 [file toxins-16-00511-s001.zip › toxins-3245296-supplementary/toxins-3245296-supplementary figures/Images/S11_alignment_Conotoxin.png]

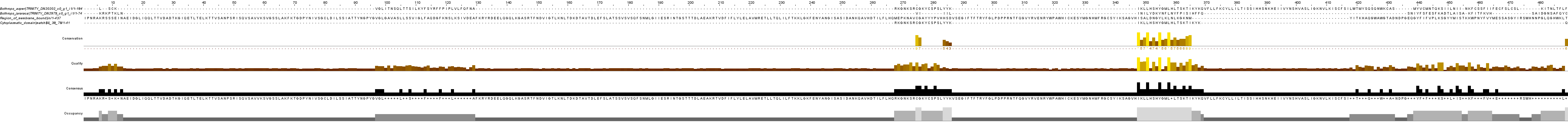

Supplement: Supplementary file 1 [file toxins-16-00511-s001.zip › toxins-3245296-supplementary/toxins-3245296-supplementary figures/Images/S12_alignment_CftxB.png]

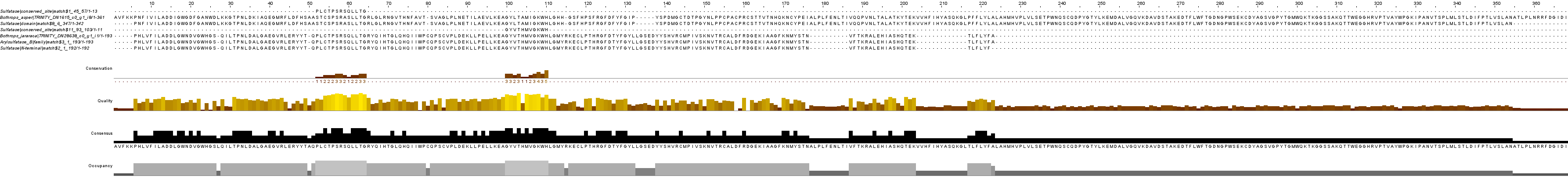

Supplement: Supplementary file 1 [file toxins-16-00511-s001.zip › toxins-3245296-supplementary/toxins-3245296-supplementary figures/Images/S13_alignment_ARS.png]

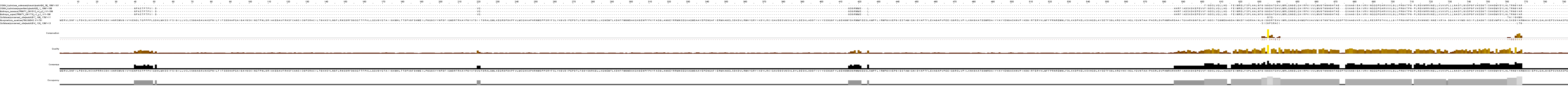

Supplement: Supplementary file 1 [file toxins-16-00511-s001.zip › toxins-3245296-supplementary/toxins-3245296-supplementary figures/Images/S14_alignment_ARS I.png]

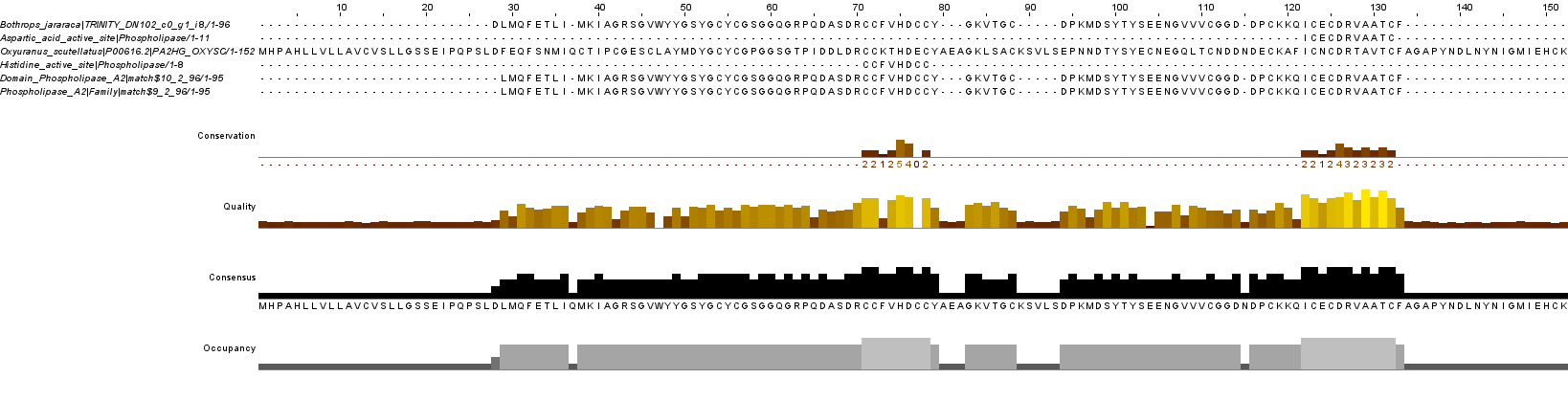

Supplement: Supplementary file 1 [file toxins-16-00511-s001.zip › toxins-3245296-supplementary/toxins-3245296-supplementary figures/Images/S1_alignment_PLA2_B.jararaca.png]

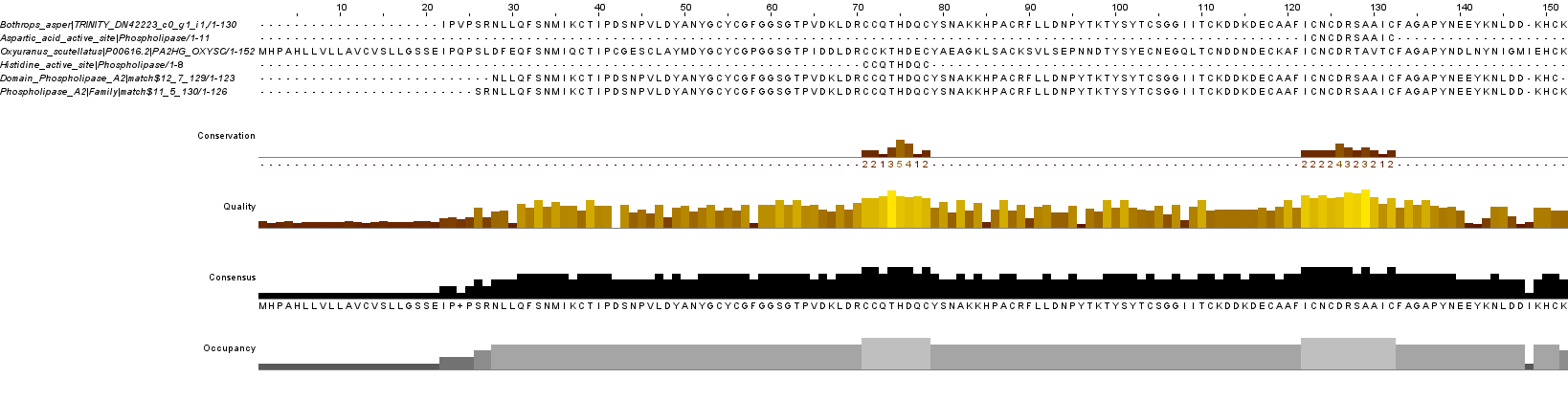

Supplement: Supplementary file 1 [file toxins-16-00511-s001.zip › toxins-3245296-supplementary/toxins-3245296-supplementary figures/Images/S2_alignment_PLA2_B.asper.png]

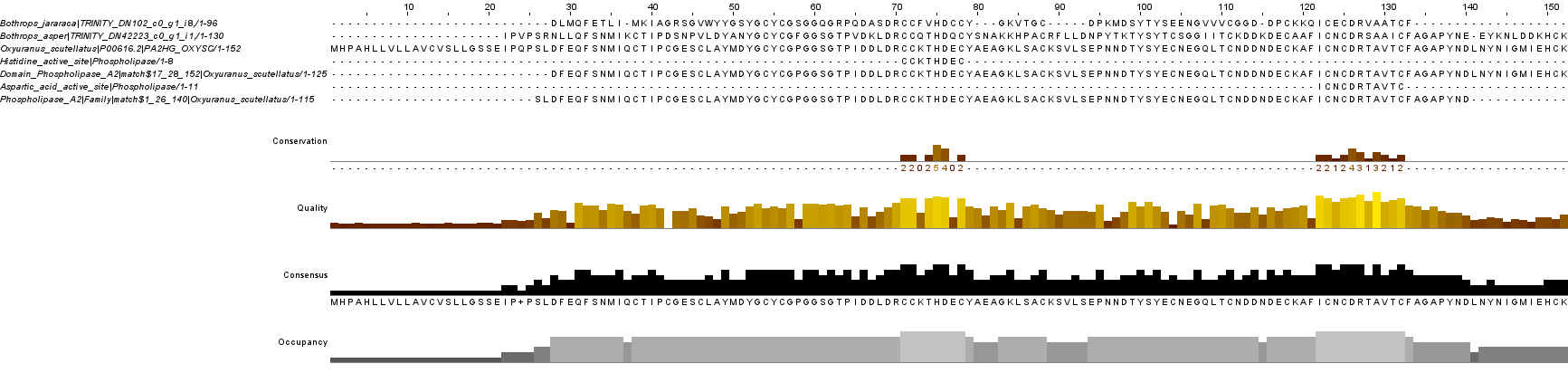

Supplement: Supplementary file 1 [file toxins-16-00511-s001.zip › toxins-3245296-supplementary/toxins-3245296-supplementary figures/Images/S3_alignment_PLA2.png]

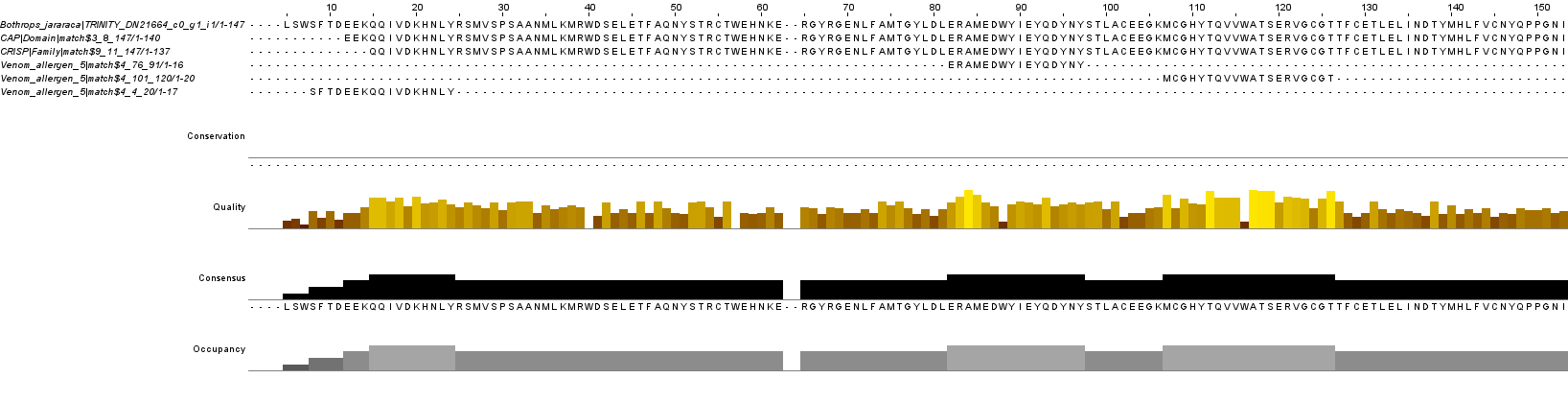

Supplement: Supplementary file 1 [file toxins-16-00511-s001.zip › toxins-3245296-supplementary/toxins-3245296-supplementary figures/Images/S4_alignment_AG5_B.jararaca.png]

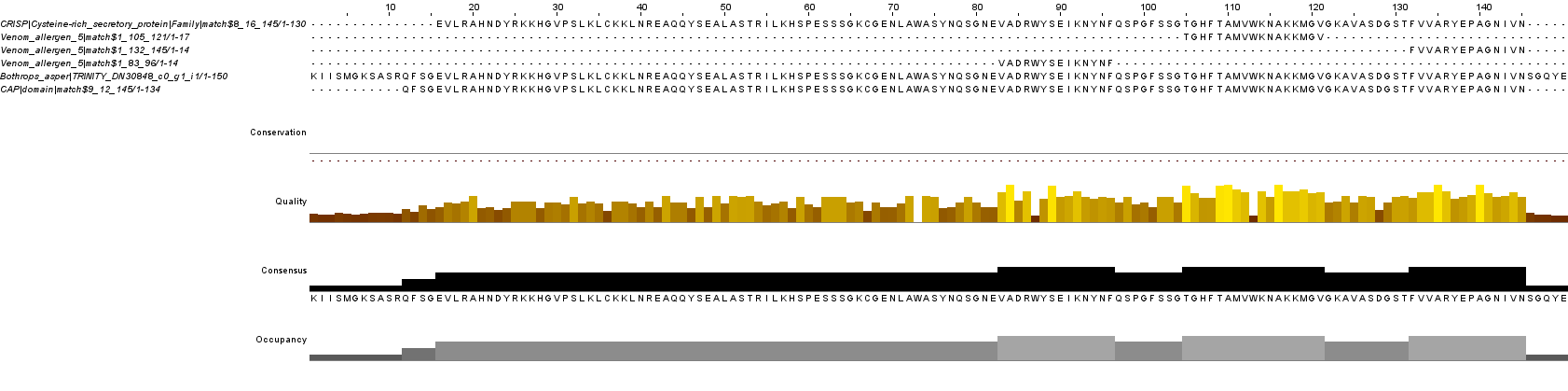

Supplement: Supplementary file 1 [file toxins-16-00511-s001.zip › toxins-3245296-supplementary/toxins-3245296-supplementary figures/Images/S5_alignment_AG5_B.asper.png]

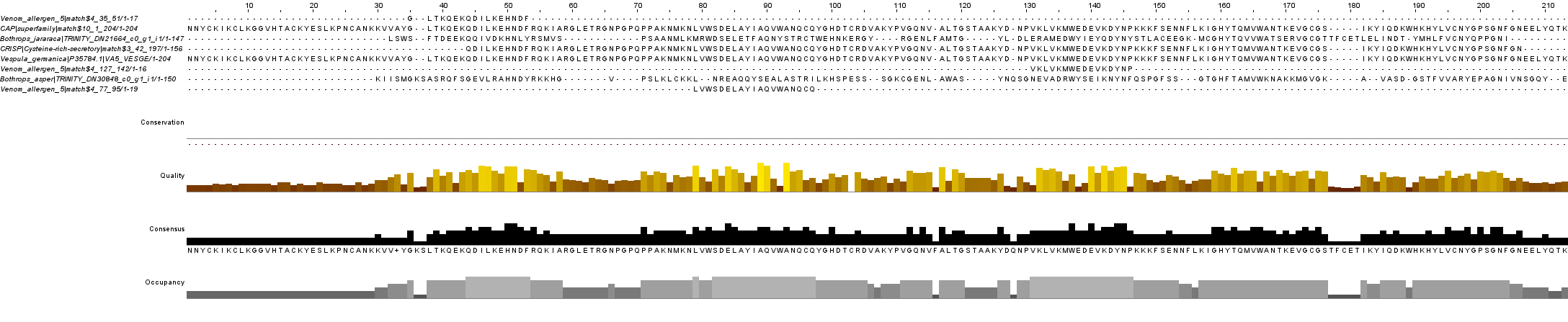

Supplement: Supplementary file 1 [file toxins-16-00511-s001.zip › toxins-3245296-supplementary/toxins-3245296-supplementary figures/Images/S6_alignment_AG5.png]

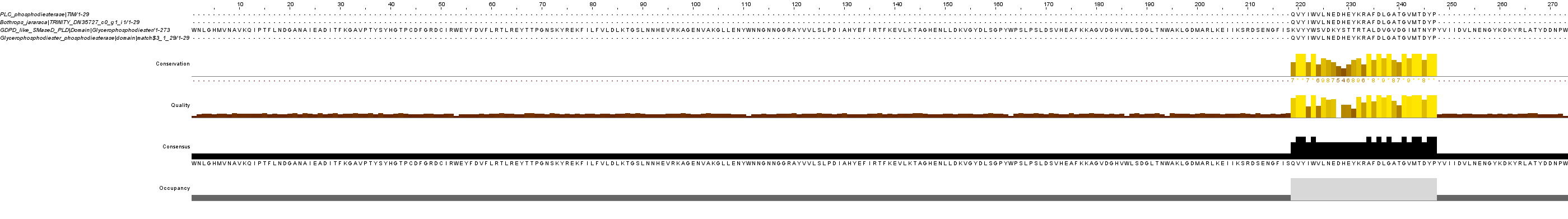

Supplement: Supplementary file 1 [file toxins-16-00511-s001.zip › toxins-3245296-supplementary/toxins-3245296-supplementary figures/Images/S7_alignment_GDPD_B.jararaca.png]

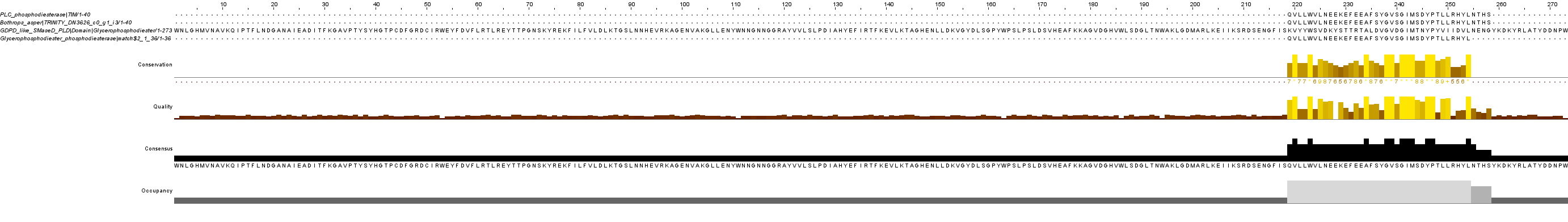

Supplement: Supplementary file 1 [file toxins-16-00511-s001.zip › toxins-3245296-supplementary/toxins-3245296-supplementary figures/Images/S8_alignment_GDPD_B.asper.png]

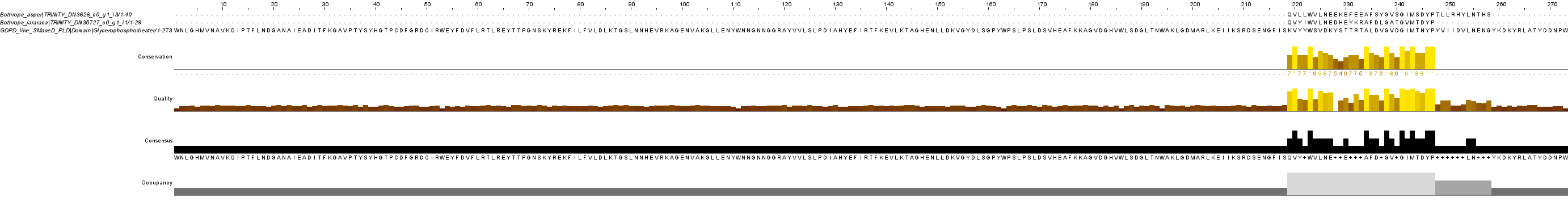

Supplement: Supplementary file 1 [file toxins-16-00511-s001.zip › toxins-3245296-supplementary/toxins-3245296-supplementary figures/Images/S9_alignment_GDPD.png]
